# Herbaceous perennial ornamental plants can support complex pollinator communities

**DOI:** 10.1038/s41598-021-95892-w

**Published:** 2021-08-30

**Authors:** E. Erickson, H. M. Patch, C. M. Grozinger

**Affiliations:** Department of Entomology, Center for Pollinator Research, Huck Institutes of the Life Sciences, Pennsylvania State University, University Park, USA

**Keywords:** Entomology, Community ecology, Urban ecology, Plant domestication

## Abstract

Human-designed landscapes can host diverse pollinator communities, and the availability of floral resources is central to supporting insect biodiversity in highly modified environments. However, some urban landscapes have relatively few pollinator-attractive plant species and management in urban environments rarely considers the function of these plants in generating and supporting a stable ecological community. Evaluations of 25 cultivars within five commercially popular herbaceous perennial ornamental plant genera (*Agastache*, *Echinacea*, *Nepeta*, *Rudbeckia*, and *Salvia*) revealed variation in the total and proportional abundance of visitors attracted. These varieties supported multiple pollinator functional groups, however bees were the primary visitors to in this system. Cultivars were assessed according to their function within a plant–pollinator network. Comparisons of artificial networks created with the six most attractive and six least attractive cultivars demonstrated that a planting scheme using the most attractive cultivars would attract nearly four times as many bee species, including several specialists and rare species. Plant diversity in the landscape was correlated with abundance and diversity of pollinator visitors, demonstrating that community context shapes a plant’s relative attractiveness to pollinators. We conclude that herbaceous perennial cultivars can support an abundance and diversity of pollinator visitors, however, planting schemes should take into consideration the effects of cultivar, landscape plant diversity, floral phenology, floral area, and contribution to a stable ecological community.

## Introduction

Insect pollinators, which play a vital role in supporting agricultural systems and terrestrial biodiversity^1^, have been experiencing global population declines^2^. A primary driver of these losses is a reduction in foraging resources due to anthropogenic land use and habitat modification^3^. However, recent studies have demonstrated that many human modified landscapes are well suited for certain pollinator taxa^4–6^. Surveys of pollinator abundance and diversity in domestic greenspaces such as gardens, parks, and green roofs suggest that these managed habitats can support abundant and diverse pollinator communities, including rare or vulnerable species^7–9^. Indeed, some authors suggest that managed greenspaces in anthropogenic landscapes may serve as ‘refuges’ for declining populations^4^ and thus should be considered as a conservation priority^10^.

Pollinator communities are complex assemblages of species with a range of nesting habits, social behaviors, nutritional requirements, and phenologies^11^. Insect species are restricted in which plant species they forage on based on preference, accessibility, and nutrient composition^12^. Some bee species collect pollen resources from one (monolectic) or a few plant species (oligolectic), while most forage broadly (polylectic)^13,14^. In co-evolved communities, such as many natural ecosystems, specialized and generalized plant and insect species interact with one another in an asymmetric—or ‘nested’- mutualism^15^. Network nestedness is a property of ecological community structure that confers stability against species loss and environmental perturbations^16,17^. Community nestedness often declines with increasing human land use^18^, so while pollinator communities in human dominated landscapes may be diverse, they are not particularly robust^19^. Increasing the availability of attractive flowering plants has been shown to be one of the most effective and reliable methods for enhancing pollinator biodiversity^20,21^, particularly in urban landscapes^22^. However, a key challenge is identifying the combinations of plant species that can support and engineer a resilient pollinator community^23,24^.

There has been a recent cultural shift towards applying ecological principles to garden design to create habitats that increase biodiversity, particularly in urban and suburban landscapes^25,26^. Furthermore, there is a marked rise in demand for pollinator-friendly plants^27,28^. Many of these biologically designed landscapes incorporate herbaceous ornamental perennial cultivars due to their naturalistic style, comparative limited breeding history, and low input cultivation requirements ^29^. Nonetheless, many of these varieties have undergone hybridization and artificial selection to create floral phenotypes based on consumer, rather than pollinator, preference^30^ and thus their utility to pollinators remains to be determined. Human selection in ornamental plants has generated a diversity of cultivars that vary in color, bloom size and duration, and morphology, all of which are important regulators of pollinator learning and choice behavior ^12,31,32^. Indeed, previous studies have found that closely related cultivars vary significantly in their attractiveness to pollinators^32–34^. Given the prevalence of cultivated ornamentals in managed urban pollinator habitats, identifying which varieties will attract a diversity of pollinator taxa and contribute to building a stable community will be integral to optimizing the ecological value of greenspaces and developing accurate recommendations for home gardeners and landscapers committed to naturalistic design.

**Figure 1:**
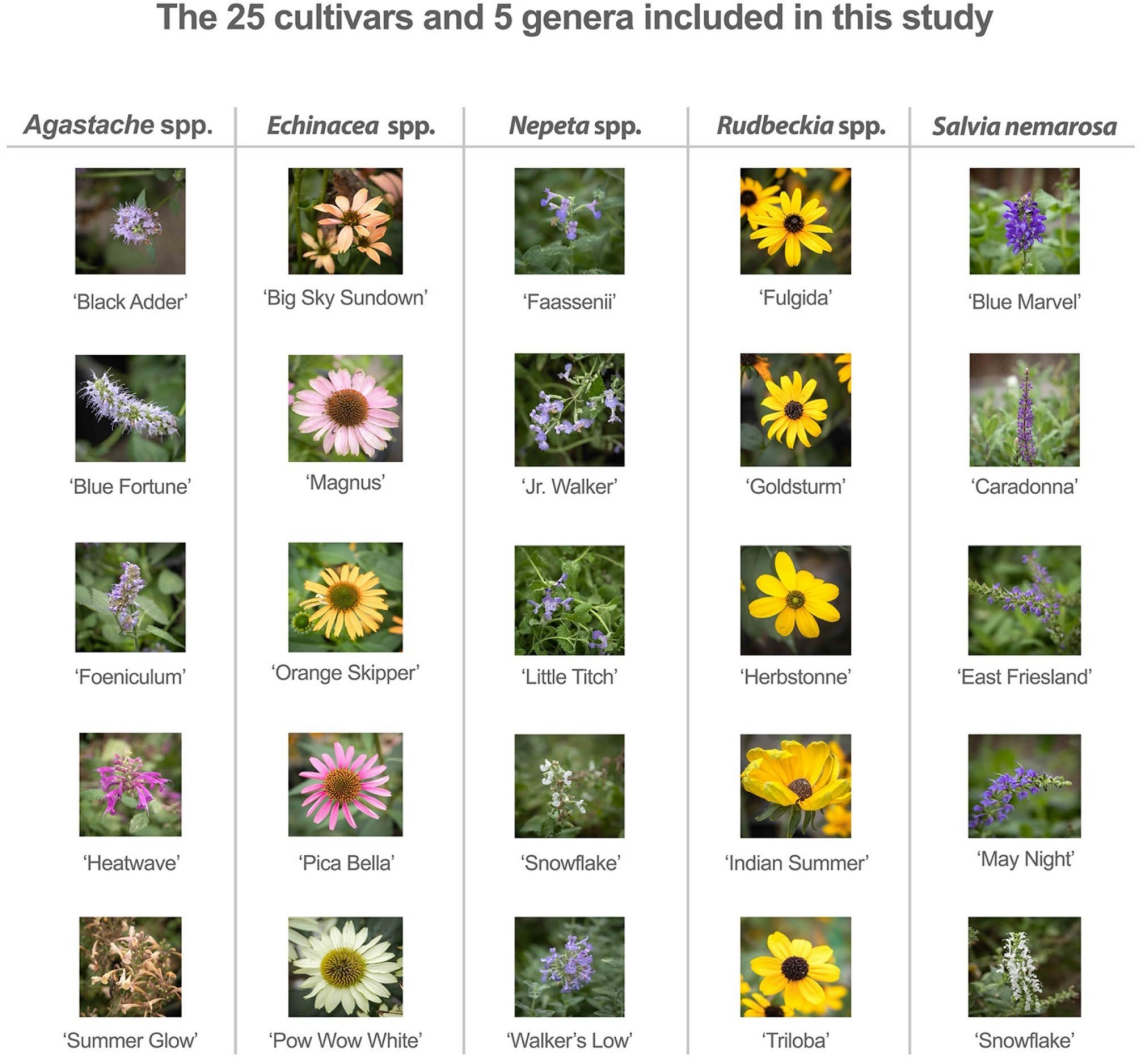
The 25 cultivars and five plant genera included in this study were selected from a 2014 NASS grower survey to reflect taxa that are commercially popular in the North American floriculture market.

We used a field-based approach to evaluate the attractiveness of 25 herbaceous perennial cultivars from five different genera that are commercially popular in North America. The genera used in this study were *Agastache*, *Echinacea*, *Nepeta*, *Rudbeckia*, and *Salvia* (Fig. 1). We evaluated attractiveness across the growing season for two years at two different sites, each of which was previously found to host a diverse pollinator population^35^. These studies allowed us to consider the value of these plants in the context of a nested and species rich ecological network and across spatial and temporal scales to provide critical insights into the cultivars and planting schemes that can best produce a resilient plant–pollinator community in human modified aesthetic landscapes.

## Results

### Abundance of visitors

**Figure 2:**
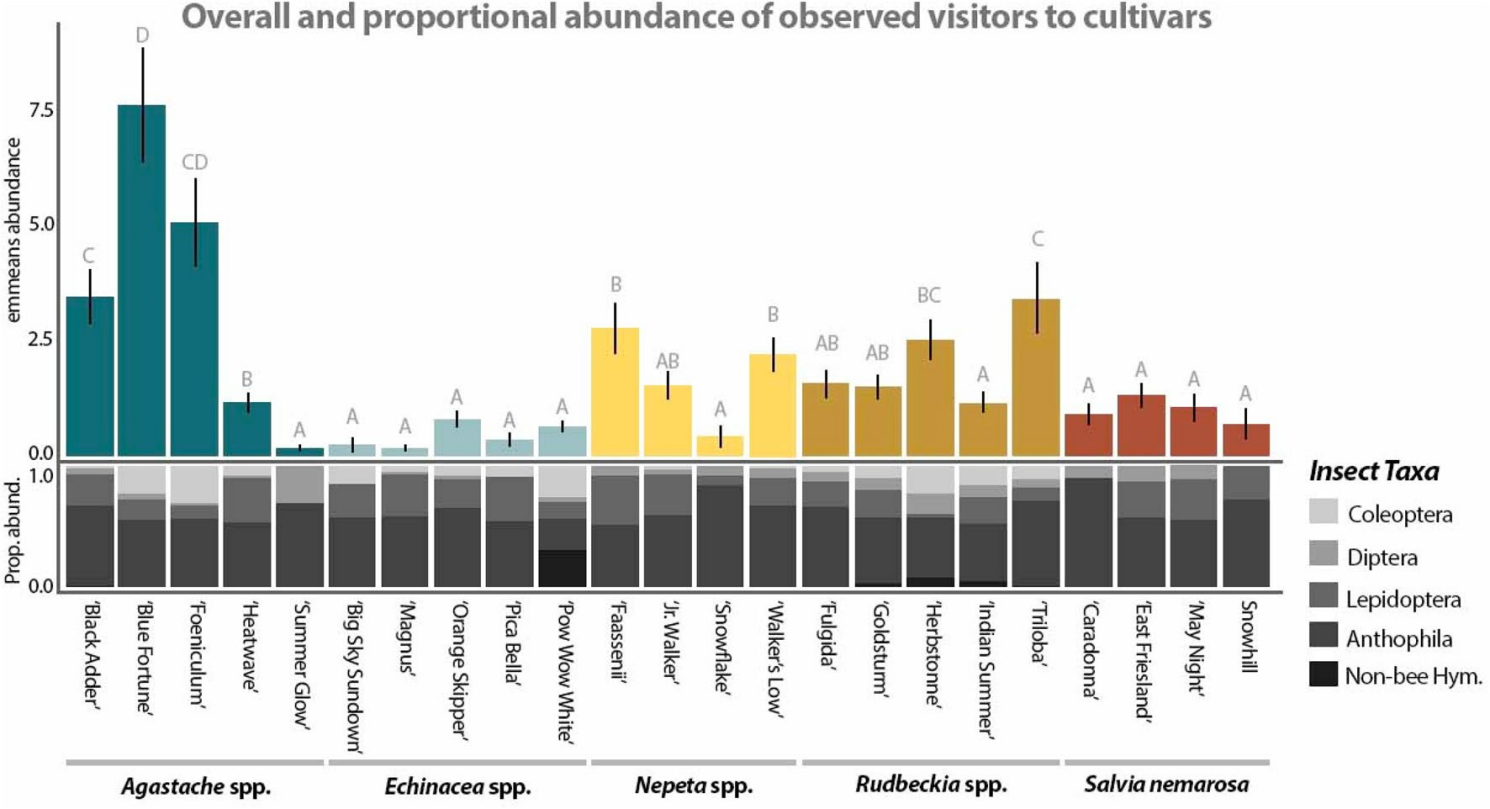
Estimated marginal means (emmeans) of total visitor abundance and mean proportional abundance of observed insect pollinator visitors. Cultivars within some genera vary greatly in total visitor abundance as well as proportional abundance of visitors, while there is little variation between cultivars of other plant genera. Anthophila species are the primary visitors observed in this system, however certain cultivars within genera attract unique pollinator functional groups.

The estimated marginal means (emmeans) of all visitors/cultivar/10 min ranged from 7.44 ± 1.22 to 0.15 ± 0.09, with *A. hybrida* ‘Blue Fortune’ receiving the most visitors and *E. purpurea* ‘Magnus’ receiving the fewest (Fig. 2). There was significant variation in the abundance of visitors attracted to cultivars of *Agastache* spp., *Rudbeckia* spp., and *Nepeta* spp. but not between cultivars of *Echinacea* sp*.* and *S. nemarosa* (Fig. 2)*.* Fourteen out of 23 cultivars differed significantly in the total abundance of visitors recorded between years, and five out of 23 cultivars differed significantly in attractiveness based on site. Consistent with other studies^36,37^, floral area was a highly significant predictor variable in the visitor abundance model (*P* < 0.001).

*Anthophila* spp. made up over half of the visitors to 22 out of 23 cultivars. Visits to *E. purpurea* ‘Pow Wow White’, however, were 26% Coleoptera species, primarily *Typocerus* spp, and 31% non-bee hymenopterans (Fig. 2).

**Figure 3:**
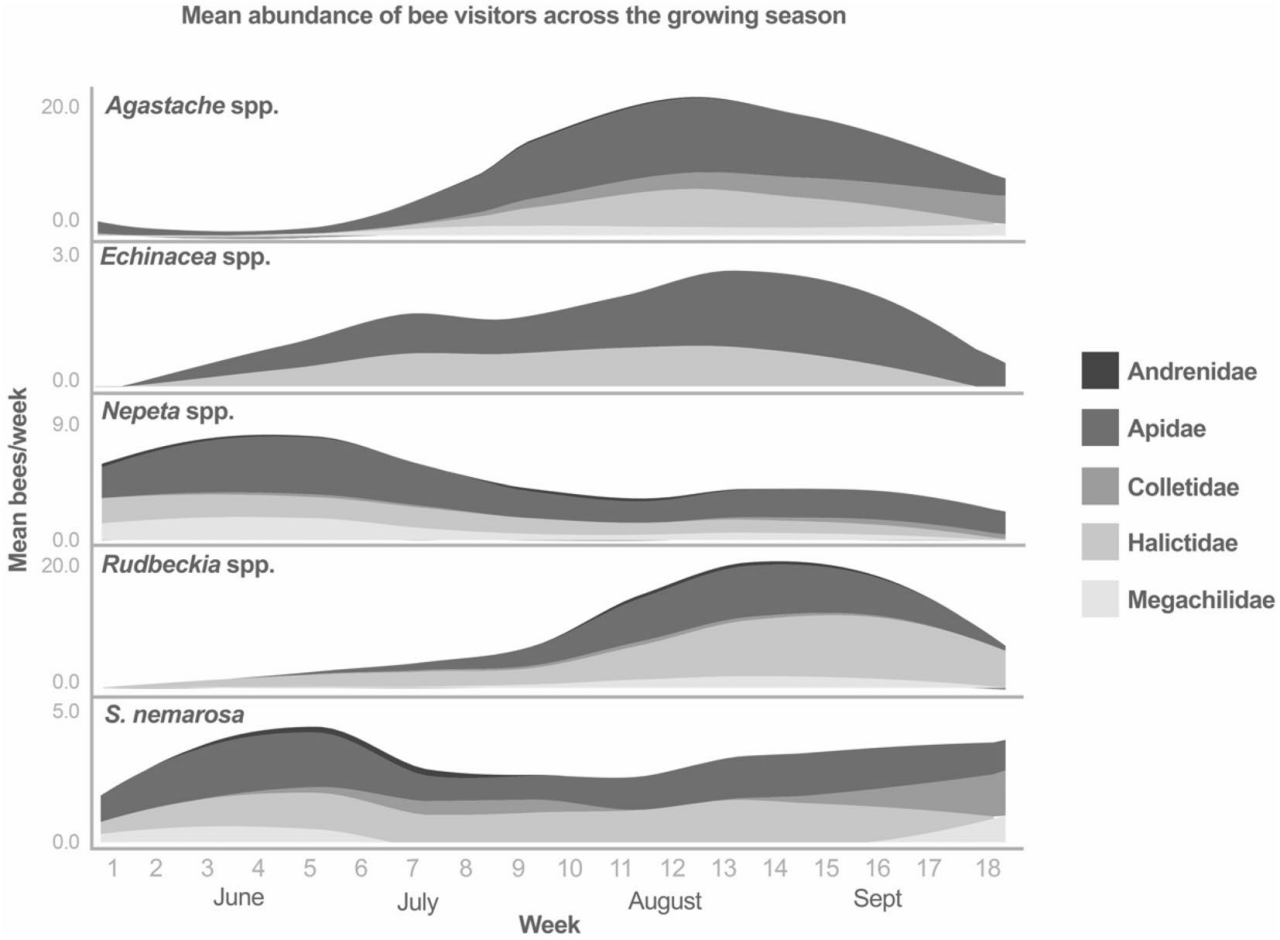
*Nepeta* spp. and *S. nemarosa* attract early season visitors while *Agastache* spp., *Rudbeckia* spp. and *Echinacea* spp. attract visitors later in the season. Seasonal patterns of bee visitation correspond closely with cultivar phenology and floral display.

The total abundance and taxonomic identity of bee visitors to the different plant genera varied seasonally. *Nepeta* and *Salvia* spp. attracted early season foragers such as Andrenidae spp. and *Bombus* spp. Between late May-mid June, the bee families visiting *Nepeta* spp. were 47.5% Apidae, 20.7% Halictidae, 22.2% Megachilidae and 9.5% Andrenidae and the families visiting *S. nemarosa* were 48.1% Apidae, 28.9% Halictidae, and 22.9% Megachilidae (Fig. 3). Visitation to *Agastache* and *Rudbeckia* spp. increased in August and September, and bee visitors to these two genera were 23.3% and 46.7% Halictidae, 20.6% and 9.7% Colletidae (genus *Hylaeus*), and 43.5% and 25.9% Apidae, respectively (primarily genera *Apis, Bombus,* and *Melissodes*).

**Figure 4:**
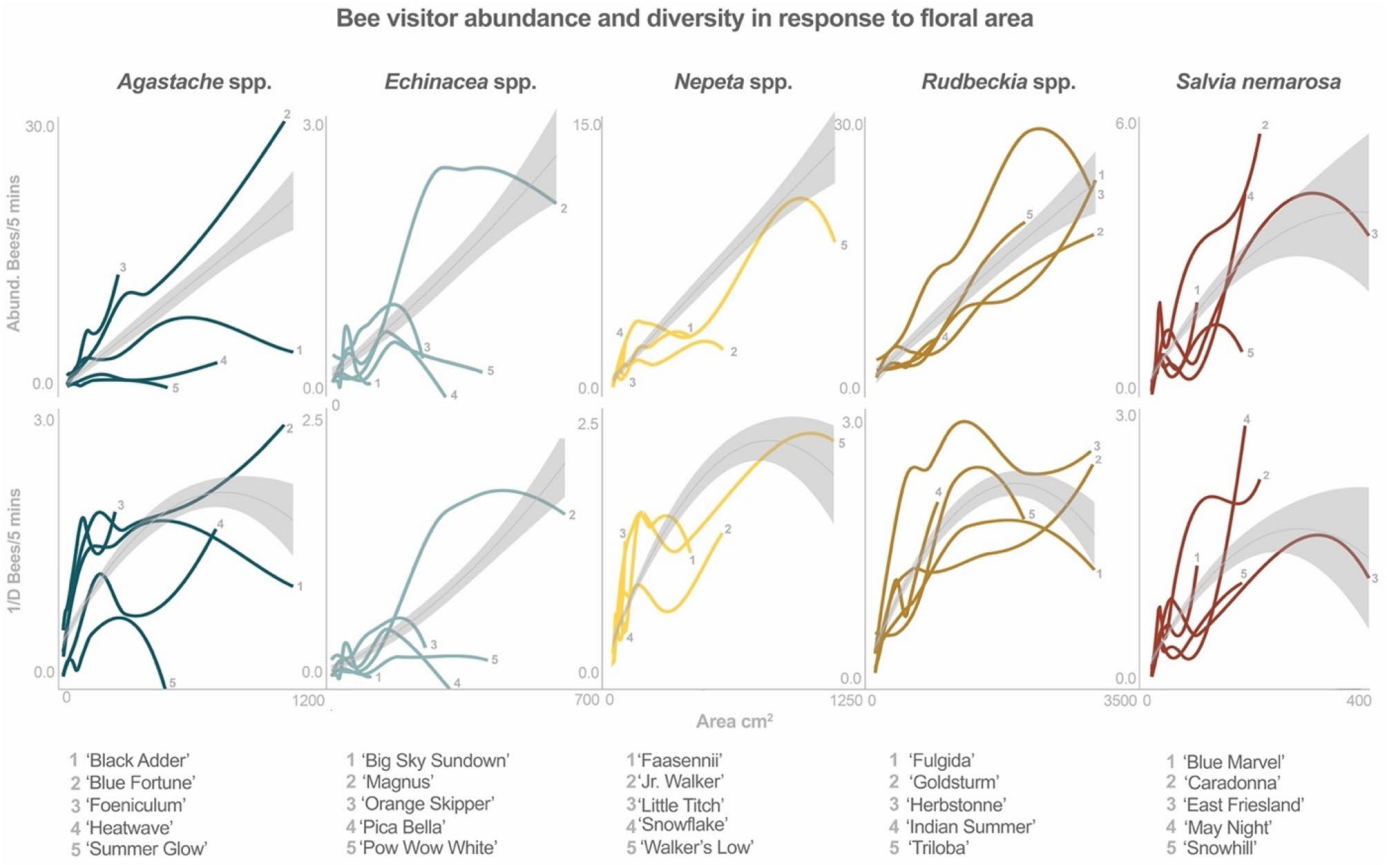
Cultivars are fit to a 'loess' regression while genera (overlaid in grey) are fit to a second order polynomial regression. For most cultivars, visitor abundance increases linearly with larger floral area while visitor diversity plateaus or decreases before peak bloom.

Seasonal patterns of bee visitation corresponded to cultivar phenology and bloom – as indicated by a positive correlation between floral area and bee visitor abundance and diversity in many cultivars (Table 1). For cultivars within four genera, bee visitor abundance increased approximately linearly with an increase in floral display area (Fig. 4). For cultivars within four genera, bee visitor diversity increased rapidly over small floral display areas then leveled off or peaked before maximum display size.

**Table 1:**
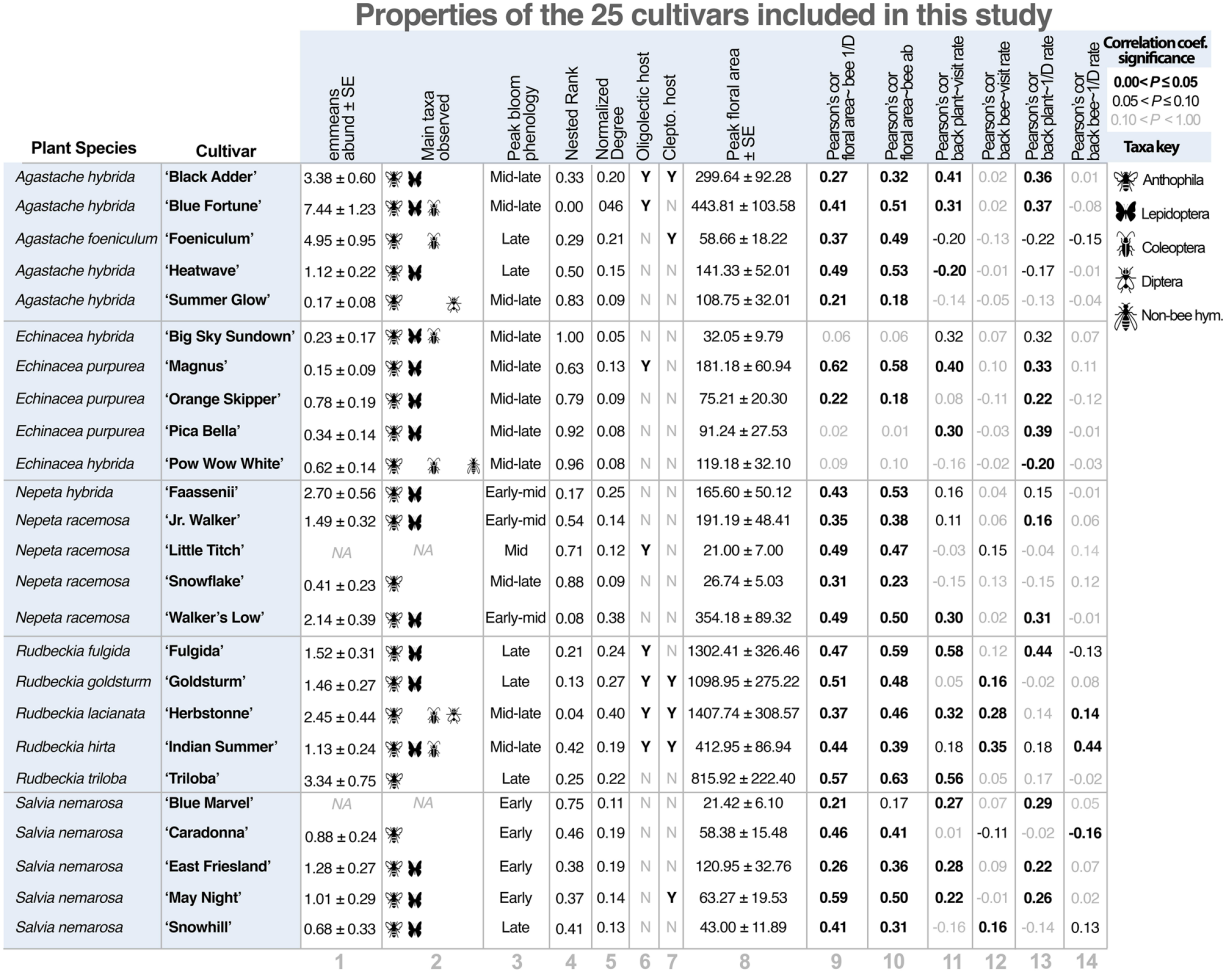
Summary of results of the quantitative analyses. **1** the estimated marginal means of total visitor abundance and **2** the primary taxonomic groups observed visiting the cultivars in this study. **3** the period during the growing season (May - September) during which the cultivar is in peak bloom. **4,5** the Nested Rank (NR) and Normalized Degree (ND) of each cultivar in a plant–pollinator network and whether that cultivar hosted **6** oligolectic or **7** cleptoparasitic bee species, based on data from snapshot collections. **8** the mean peak floral display area for each cultivar. Pearson's correlation coefficients and the significance of correlation tests between floral display area and **9,10** bee diversity and abundance. Pearson's correlation coefficients and the significance of correlation tests between bee abundance (**11,12**) and diversity (**13,14**) rates (standardized by floral display area) and background plant and bee diversity.

### Effect of landscape variables on plant attractiveness

Background plant and insect diversity differed significantly between sites (*P* < 0.001) with Site 2 having a higher diversity of both plants and bees (see Supplementary Material for species). At both sites, background bee and plant 1/D were significantly positively correlated with each other (cor = Site 1: 0.23, Site 2: 0.17, *P* < 0.001) and were significantly negatively correlated with week (Bee cor = Site 1: − 0.63, Site 2: −0.32, *P* < 0.001; Plant cor = Site 2: − 0.27, *P* < 0.001) with the exception of plant diversity and week at Site 1 (cor = − 0.02, *P* = 0.23). Across sites, background plant 1/D had a significant positive (*P* ≤ 0.05) or close to significant (0.05 < *P* ≤ 0.10) effect on the 1/D/Area cm^2^ (1/D ‘rate’) of bee visitors to 14 cultivars and a negative effect on three cultivars. Plant 1/D had a positive effect on visitor abundance/area cm^2^ (visitation rate) to 15 out of 25 cultivars and a negative effect on two cultivars. Background bee 1/D had positive effect on bee visitor 1/D ‘rate’ to three cultivars and negative effect on two cultivars. Background bee 1/D and had a positive effect on bee visitation rate to five cultivars and a negative effect on one cultivar (Table 1).

### Network properties

**Figure 5:**
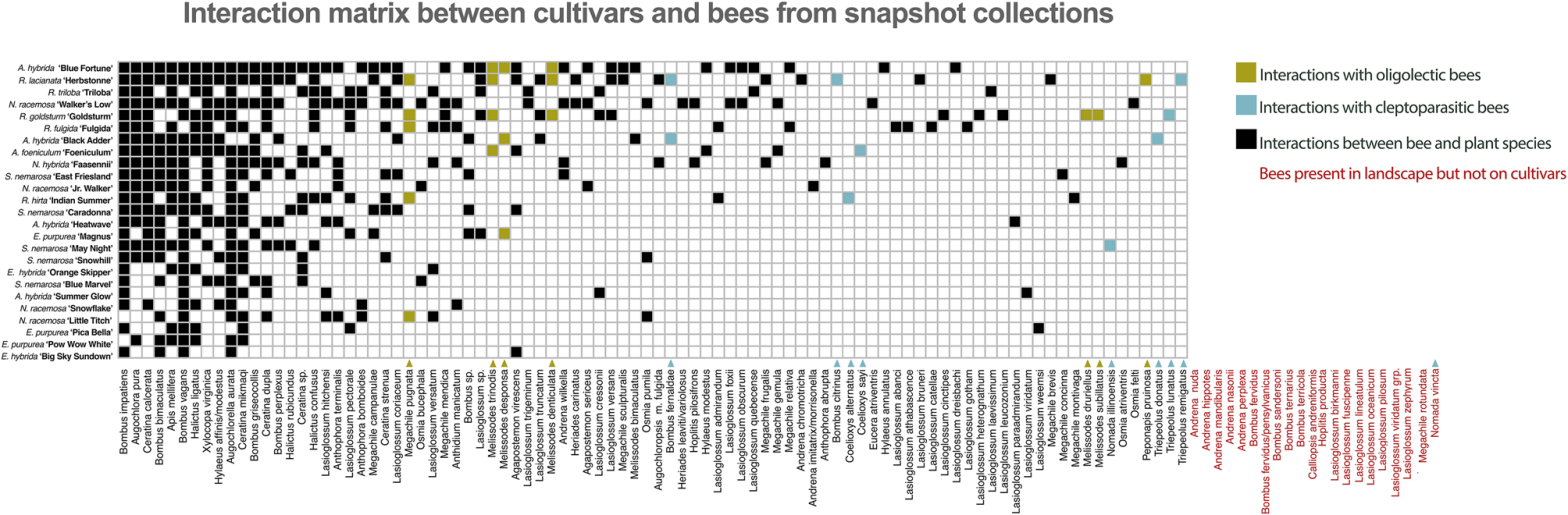
Cultivars are on the vertical axis and bee species (snapshot and background collections) are on the horizontal axis. The cultivars in this study interact with a diversity of species, including uncommon dietary specialists and cleptoparasitic species. Certain cultivars have the potential to contribute to community nestedness.

Of the 106 bee species identified at our study sites, 86 were found to visit the plant cultivars. Of the 86 bee species collected on cultivars, only 39 were collected in traps. Seven species collected on cultivars in this study were classified as oligolectic (*P. pruinosa, M. subillatus, M. druriellus , M. trinodis, M. desponsa, M. denticulata, M. pugnata*) in previous studies^11,38–40^. Eight out of nine total cleptoparasitic species present in the landscape were collected on cultivars (*T. remigatus, T. lunatus, T. donatus, N. illinoensis, C. sayi, C. alternatus, B. fernaldae,* and *B. citrinus*)^41–43^ (Fig. 5). *Lasioglossum dreisbachi*, which was found on *A. hybrida* ‘Blue Fortune’, is a state record for Pennsylvania.

We calculated species-level and network level properties to assess the potential for these cultivars to support a complex ecological community. Nested Rank (NR) is the ranked functional importance of a species in a mutualistic network arranged to maximize nestedness ^44,45^ where species' with value 0 are most important. Normalized Degree (ND) describes the number of interacting partners a species supports in relation to the total possible partners in a community^46^, regardless of the taxonomic identity or functional role of those partners. ND indicates a species' generalized behavior in a community whereas NR is a reflection of its contribution to sustaining a resilient network^44^.

The cultivars with the highest ND (interacted with the most bee species) were *A. hybrida* 'Blue Fortune' (ND = 0.46), *N. racemosa* 'Walker's Low' (ND = 0.38), and *R. lacianata* 'Herbstonne' (ND = 0.40). The cultivars with the lowest ND were *Echincaea* spp. ‘Big Sky Sundown’, 'Orange Skipper', 'Pica Bella' and 'Pow Wow White (NDs = 0.05, 0.09, 0.08, 0.08) and *N. faassennii* ‘Snowflake’ (ND = 0.09) (Table 1). The most generalized bee species collected on plants included *B. impatiens* (ND = 0.92), *B. vagans,* (ND = 0.92), and *A. aurata* (ND = 0.88)*.*

Plant species with low NR have the greatest contribution to nested community structure. In this study, cultivars with low NR interacted with many bee taxa in a network, including rarer oligolectic and cleptoparasitic species, while those with high NR interacted with few, primarily abundant generalist, species. The species with the lowest NR were *A. hybrida* ‘Blue Fortune’ (NR = 0.00), *Rudbeckia* spp. ‘Herbstonne’, ‘Goldsturm’, ‘Fulgida’ and ‘Triloba’ (NRs = 0.04, 0.13, 0.21, 0.25), and *Nepeta* spp. ‘Walker’s Low’ and ‘Faassennii’(NRs = 0.08, 0.17) and the cultivars with the highest NR were *Echinacea* spp. ‘Big Sky Sundown’, ‘Pow Wow White’ and ‘Pica Bella’ ( NRs = 1.00, 0.96, 0.92) and *N. racemosa* ‘Snowflake’ (NR = 0.88) (Table 1).

Nested rank was significantly negatively correlated with mean non-zero floral display area (cor = − 0.63, *P* < 0.001), and normalized degree was significantly positively correlated with non-zero floral area (cor = 0.54, *P* = 0.01)—meaning cultivars with larger floral displays supported more species and had the greatest contribution to nested community structure.

**Figure 6:**
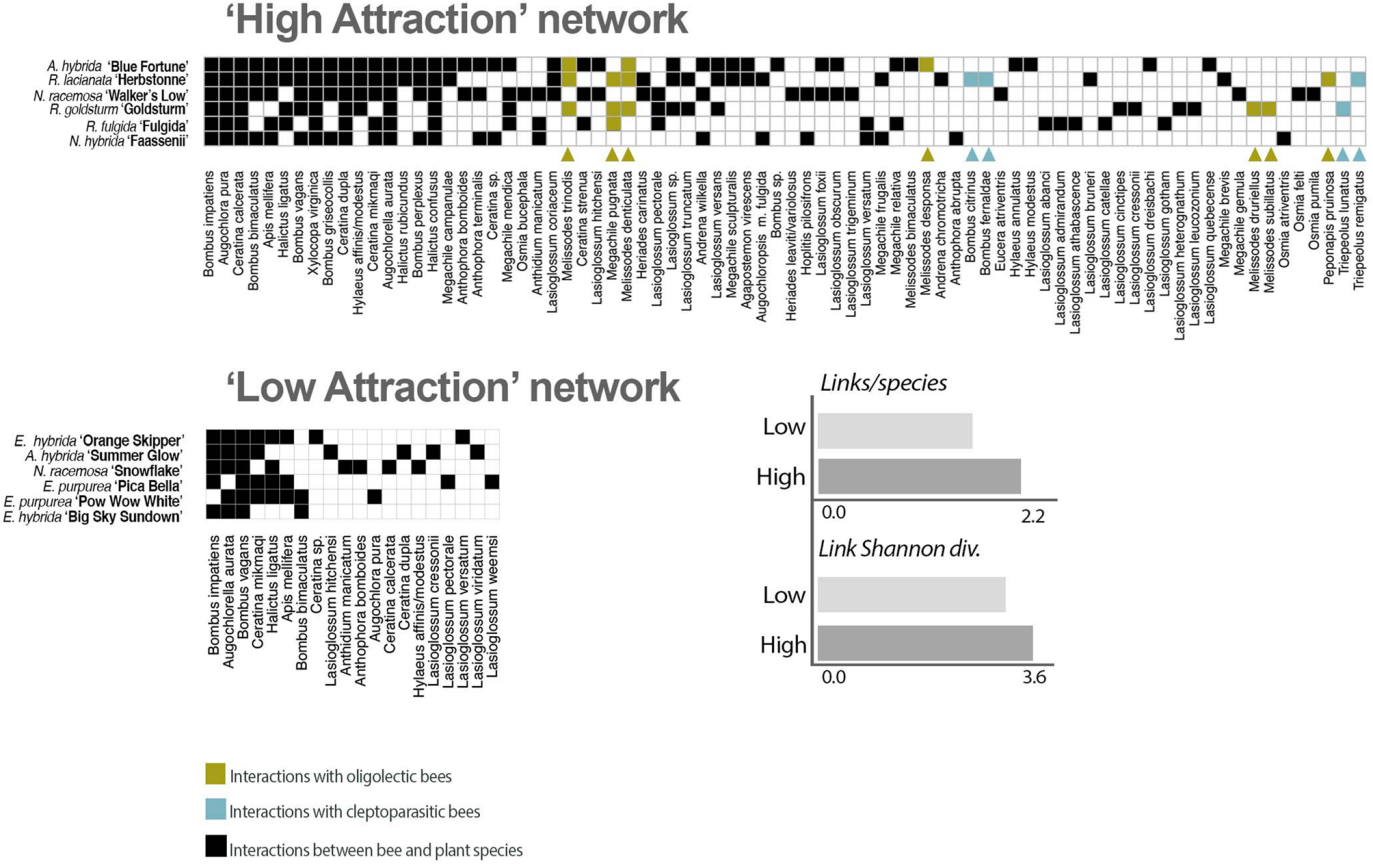
Hypothetical networks are constructed with 'high' and 'low' attraction cultivars, based on ND and NR. Domestic landscapes planted with 'high attraction' cultivars are capable of supporting a greater abundance and diversity of species, including dietary specialists and cleptoparasites whereas landscapes planted with 'low attraction' cultivars will support few species and exhibit relatively low functional redundancy—similar to unstable random communities as described in Lever et al.^80^.

To estimate the network structure of a hypothetical landscape planted with these varieties, we calculated network properties for two subset communities containing the six cultivars with the lowest nested ranks and highest normalized degrees ('high attraction') and six cultivars with the highest nested rank and lowest normalized degree ('low attraction'). The 'high attraction' cultivars were *A.hybrida* 'Blue Fortune', *Nepeta* spp. 'Walker's Low' and 'Faassennii', and *Rudbeckia* spp. 'Herbstonne', 'Goldsturm' and 'Fulgida'. The 'low attraction' cultivars were *Echincaea* spp. ‘Big Sky Sundown’, 'Orange Skipper', 'Pica Bella' and 'Pow Wow White', *N. faassennii* ‘Snowflake’, and *A. hybrida* 'Summer Glow'. The network with six 'high attraction' cultivars supported 78 total pollinator species, including four cleptoparasitic species and all seven oligolectic species with an average of 2.15 links/species and a link Shannon diversity of 3.61. The network with the 6 'low attraction' cultivars supported only 20 bee species, all of which were polylectic with an average 1.62 links/species and a link Shannon diversity of 3.42 (Fig. 6).

## Discussion

This study demonstrates that many herbaceous ornamental perennial cultivars are capable of supporting an abundance and diversity of pollinator taxa, including relatively uncommon cleptoparasitic species and dietary specialists. These cultivars may serve as generalist ‘hubs’ within a plant–pollinator network and will disproportionately contribute to a resilient nested community structure by providing nutritional resources to a range of insect functional groups (see Olesen et al. 2007 for discussion^47^). Consistent with other studies^33,34^, we found that cultivars varied significantly in their bloom times, their level of attractiveness in terms of visitor abundance, the diversity of functional groups that they attracted, and their robustness to spatial and temporal variation in environmental conditions and the surrounding plant and insect community (Table 1). These factors should all be considered when selecting cultivars for managed pollinator habitat.

A number of the cultivars that attracted insect functional groups unique from other cultivars in their genera, such as *E. purpurea* ‘Pow Wow White’ and *A. hybrida* ‘Summer Glow’, are varieties that vary notably from the phenotype of their parent species. This indicates that cultivar development may create new combinations of floral traits that attract different pollinator taxa. There are concerns that selective breeding for aesthetic value to humans may have reduced the attractiveness and nutritional value of ornamental plants for pollinators^31,48,49^. Indeed, certain cultivars in this study had reduced attractiveness compared to other varieties within genera, which may be due to variation in traits such as floral color^50^, morphology^51^, or display size^36^. Further studies are needed to examine the relationship between multimodal cultivar phenotype and pollinator choice behavior. Nonetheless, most cultivars included in this study were moderately to highly attractive to pollinator visitors, indicating that for many perennial ornamentals, breeding has not constrained their accessibility to pollinators and the pollinator attracting phenotype is likely maintained.

Many of these varieties are selected for naturalistic traits^52^, and thus floral advertisement may be a sufficiently honest signal to pollinators, including more specialized species^48,53^. Cultivars of *Agastache* spp., *Rudbeckia* spp., and *Echinacea* spp. are native to the Nearctic and attracted the most oligolectic species overall, likely reflecting co-evolved relationships in plant–pollinator networks. Notably, *N. racemosa* ‘Little Titch’ which is non-native to the Nearctic^54^, attracted the oligolectic species *Megachile pugnata.* However, *M. pugnata* is described as a pollen specialist on Asteraceae^38^, suggesting that visitors were collecting nectar resources^55^.

Pollinator species vary in the time of year when they emerge and provision their nests^56^. Thus, a complete pollinator habitat will include flowering plants with overlapping phenology to ensure a consistent availability of foraging resources^57,58^. The five herbaceous perennial ornamental plant genera included in this study varied in the abundance of visitors attracted across our temperate seasons primarily based on difference in phenology and peak bloom period (Table 1). Although cultivars of some genera, such as *Salvia* and *Nepeta* spp. are not highly attractive overall (Fig. 2), they can play an important role by providing foraging resources early in the season (Fig. 3), particularly when paired with other high-bloom spring resources such as flowering trees^59,60^. While there is a distinct temporal component to a cultivar’s attractiveness, ornamental plants are often selected for an extended bloom time^33,61^, making them well suited for providing nutritional resources during seasonal dearth periods and ideal for use in a successional garden.

Bee communities respond positively to increases in host plant availability and diversity^62,63^, and patterns of pollinator visitation to plants is often dependent on community context^45,64^. We found that differences in background plant diversity across the season had a measurable, often positive effect on cultivar attractiveness. This supports findings from other studies that pollinator activity increases with flowering plant density and diversity in the landscape^63^. It is notable that visitation to most of the cultivars included in the study (19 out of 25) were not affected by variation in background insect communities, although background bee and plant communities were positively correlated. Previous studies have noted that it is challenging to assess the background insect community because blue vane and bowl trapping methods tend to be biased towards smaller bodied bees^65^, and, if floral diversity is high, bees are more likely to be found on flowers than in artificial traps^66^.

We found that floral display area was a consistent predictor of plant attractiveness and ecological utility to pollinators across multiple analyses. For some cultivars and genera, the relationship between the abundance of bee visitors and floral area was approximately linear (Fig. 4). This is not the case in other cultivars, which may be due to direct competition with other functional groups in the landscape, such as flies, that preferentially forage on larger floral displays^67^. Bee visitor diversity tended to increase rapidly over relatively small changes in floral area until leveling off to a more gradual rate or even decreasing at larger floral display sizes. These results reinforce the adage of ‘the more flowers the better’ when creating habitat for pollinators^68^ and the value of providing species rich floral resources to support a diverse community.

Many of the previous studies on the interactions between ornamental plants and pollinators have been conducted in urban and suburban landscapes, and have concluded that most cultivars of herbaceous ornamental plants species that have been tested are poorly attractive^32,34,69^. Urban pollinator communities are shaped by unique landscape characteristics such as habitat fragmentation, prevalence of invasive species, and urban warming^3,70^ and tend to disproportionately comprise dietary and habitat generalists^71^. By testing the attractiveness and function of these cultivars in a seminatural and species rich landscape, we can identify varieties that are amenable to domestication and may bring in vulnerable species and help build more resilient communities in highly disturbed human-dominated habitats.

We adapted ecological network theory and analysis to identify cultivars that may be used to design a resilient and robust plant–pollinator community in constructed and managed landscapes (i.e. gardens, parks, verges). Even in a landscape with a well-established and nested plant–pollinator community, many varieties had a high ND and low NR—indicating that they may play a fundamental role in supporting a nested and stable community structure. Indeed, a theoretical landscape planted with these high attraction cultivars would be capable of hosting a range of pollinator taxa and functional groups. Notably, many cultivars that were most attractive overall were also those that had the greatest contribution to maintaining community stability - suggesting that in herbaceous ornamental perennial species, visitor abundance may be a suitable proxy for ecological function. However, further studies are needed to test these hypotheses in a field setting.

We also found that a plant’s potential to serve as a generalist host plant to a nested pollinator community was positively correlated with floral display size, such as with cultivars of *Rudbeckia* spp. and *Agastache* spp. These cultivars have a higher likelihood of attracting and provisioning rarer and more vulnerable species and may therefore be valuable candidates for planting multiply in a pollinator garden. Other species with comparatively small floral displays and low generality, such as cultivars of *S. nemarosa* and *Echinacea* spp., can be planted more sparingly while still contributing to overall floral diversity and abundance in the landscape. An understanding of the relationship between ecological function, plant attractiveness, and floral display size may be applied to garden design—particularly in areas with spatial limitations.

## Conclusion

Herbaceous perennial ornamental flowering plant species are popular among home gardeners and landscape designers and can support an abundant, diverse, and resilient pollinator community. There is considerable variation among cultivars – including overall attractiveness and attractiveness to certain insect functional groups, contribution to a nested network, and phenology (see Table 1). With thoughtful consideration of this variation, ornamental herbaceous perennials can be valuable tools for creating ecologically resilient and aesthetically pleasing pollinator habitat in human modified landscapes.

## Methods

### Plant selection

Plants were selected based on wholesale value from a 2014 NASS grower survey^72^. We identified five herbaceous perennial genera that are popular in the Northeastern PA market (S. Adam, Pennsylvania State University Extension, Personal Communication), and had overlapping and multi-week blooms. The plant taxa used in this study were: *Salvia nemarosa*, *Nepeta* spp., *Echinacea* spp., *Rudbeckia* spp., and *Agastache* spp. Additionally, we selected five cultivars of each plant genus (Fig. 1) representing a range of floral phenotypes. Plants were purchased in 2017 from North Creek Nursery (Oxford, PA), Creek Hill Nursery (Leola, PA), Russell Gardens (Southampton, PA), Morningsun Farm (Vacaville, CA) and Bluestone Perennials (Madison, OH). All plants were purchased and managed in compliance with local and national regulations. Plants were assessed annually for overwinter survival and replaced when needed.

### Plot design

Field observations were conducted at two sites at the Penn State Russell E. Larson Agricultural Research in Pine Grove Furnace, PA (Site 1 = 40.704634, − 77.973045, Site 2 = 40.712329, − 77.933609) that were located in a semi-natural agricultural landscape on a forest edge and hosted a nested and diverse plant–pollinator network^33,35^. The sites are ~ 3.5 km apart, so the pollinator communities were likely independent^73^ while climatic variables were consistent. At both sites, plants were arranged in a randomized complete block design with four blocks per site and one replicate of each cultivar per block, for a total of 100 plants at each site. Blocks were separated by 1.5 m borders; plants within blocks were spaced 1 m apart (see Supplementary Material for plot design).

In 2018, plants were placed directly in the ground at Sites 1 and 2. Site 1 has a clay rich soil and floods regularly, resulting in poor plant growth and survival. Thus, in 2019, plants were in pots at Site 1. Plants at Site 1 were potted in five gallon pots using Sungro MetroMix 830 (Agawam, MA) media. Plants at both sites were fertilized at the start of each season to genus specific levels (see Supplementary Material for fertilization rates) with Osmocote Plus 7.5 g Tablets 15-8-11 (Scott’s Miracle-Gro, Marysville, OH). Plants ranged in peak height from 2.5 cm (*S. nemarosa* 'May Night' to 228 cm (*R. lacianata* 'Herbstonne').

### Pollinator observations and snapshot collections

Pollinator visitation was recorded by two observers (R. Kaneshiki and E. Erickson) in 2018 and one observer (E. Erickson) in 2019. To account for variation in daily pollinator activity cycles^74^ the order of observations was randomized for each data collection session. All plants were observed weekly in sets of four for 10 min once between the hours of 9:00 and 13:00 (AM) and once between 13:01 and 17:00 (PM). Observations were recorded throughout the duration of bloom from June 7 to September 12, 2018 and May 21 to September 11, 2019. Each pollinating insect that visited the focal plant during the observation period was identified to morphotaxa (See Supplementary Material for groupings). Dates of observations and collections were standardized across years using accumulated growing degree days.

We conducted bi-monthly ‘snapshot’ collections using an Insect Vacuum (BioQuip, Rancho Dominguez, CA) to collect all pollinating insect visitors to each plant for five minutes. Only Anthophila specimens were ultimately included in the analysis. These collections were also divided into ‘AM’ and ‘PM’. Collections were done row wise and in sets of two with the start point alternated for each data collection event. Snapshot collections ran from June 5 to September 28, 2018 and from May 23 to September 23, 2019. Specimens were euthanized in the field using dry ice then transferred to individual vials and stored in the laboratory at − 20 °C. Specimens were pinned and bees identified to species by Sam Droege (U.S. Geological Survey).

### Characterization of background plant and insect community

We performed bi-monthly modified transect samples to characterize the blooming flower species in the landscape surrounding Sites 1&2. For each site, we randomly selected 10 out of 40 possible starting locations along the perimeter of the plot. Transects started at each of 10 selected locations and total number of flowers for each species was estimated by E. Erickson counting all plants within a 0.9 m radius at five equally spaced points along the transect. Distances between sampling points were between 1.25 and 6 m and were randomly selected for each transect. Vegetation sampling was performed in 2019 only, however the non-crop space (100% at Site 1 and ~ 75% at Site 2) consists of undisturbed and unmanaged habitats and the portion of the landscape used for crop production (25% of Site 2) was planted each year with corn, and thus results should be comparable across years.

Assessments of background insect diversity were performed bi-monthly using blue vane traps (with a 64 oz vessel) and white, yellow, and blue 3.25 oz bowl traps (NHSSI, Upper Marlboro, MD). Vane traps were mounted 1 m off the ground and bowl traps were elevated to the level of vegetation using adjustable supports^75^. Two sets of each style of trap were mounted opposite each other at the perimeter of the research plot at each site. Traps were filled with soapy water and left for 24 h on days with low wind and little chance of rain. Specimens were extracted using a nylon strainer, suspended in 70% ethanol, and stored in plastic sample bags (Whirl–Pak, Madison, WI). Specimens were washed, dried, and pinned in the lab and Anthophila species were identified by S. Droege.

### Floral area estimation

Each week, we photographed plant replicates from above while holding a measuring stick at the crown of the foliage. Photos were processed in Photoshop (Adobe 18.1.1) by setting a unique value for pixel number/cm on the measuring stick and selecting all pixels with open flowers to calculate bloom area cm^2^.

### Quantitative analysis

#### Abundance

All statistical analyses were performed in R 3.6.1^76^. A generalized linear nixed effects model (GLMM) fit to a Poisson distribution was used to model the effects of predictor variables on the response variable ‘total visitor abundance’ from field observations (see Supplementary Material for model). All observations on plants with a floral display area smaller that 5 cm^2^ were omitted and observations on *N. faassennii* ‘Little Titch’ and *S. nemarosa* ‘Blue Marvel’ were excluded from analyses due to poor plant growth and low replication. The model was selected based on AIC and residuals. The estimated marginal mean value for each cultivar was extracted using the ‘emmeans’ package^77^ and pairwise comparisons of interaction effects were calculated using a Tukey post-hoc adjustment.

The proportional abundance of visitors by functional group was calculated by Averaging replicates across cultivars. Visitation by bee taxa from snapshot collections across the season were estimated by averaging the sum visitors/bee family/week/replicate across cultivars.

#### Effect of landscape variables

The sum abundance and inverse Simpson’s Diversity (1/D) of bee specimens from the snapshot collections was calculated for each plant replicate with a floral area greater than zero for each collection event using the ‘Vegan’ package^78^. These values were standardized by dividing by floral area to calculate diversity or abundance of visitors/cm^2^/5 min. Visitor abundance and diversity rates for each cultivar were tested for correlation with 1/D of bee samples from traps and the 1/D of the background plant surveys using the Pearson's correlation coefficient. 1/D of bee specimens collected in traps was tested for correlation with background plant 1/D using a Pearson's correlation coefficient and were compared between sites using a one-way ANOVA.

#### Floral area

The relationship between non-zero floral area and bee visitor abundance and diversity from each snapshot collection event was visualized using a ‘loess’ regression for cultivars and a second order polynomial linear model for genera in the ‘ggplot2’ package^79^ (Fig. 6). Correlation between bee visitor 1/D and abundance (from snapshot samples) and floral display area was estimated for each cultivar across years and sites using a Pearson's correlation coefficient.

#### Plant–pollinator network properties

Nested rank (NR) and normalized degree (ND) of the summed interactions between bee species from snapshot collections and cultivars, and network level properties of hypothetical subsets of cultivars were analyzed using the ‘bipartite’ package in R^46^. Correlation between NR, ND and non-zero floral area was tested using a Pearson's Correlation Coefficient.

## Supplementary Information

Supplementary Information.

## Data Availability

All data and R scripts relating to this research have been made publicly available via Dryad 10.5061/dryad.s4mw6m96s.
