# Author Correction: Herbaceous perennial ornamental plants can support complex pollinator communities

**DOI:** 10.1038/s41598-022-07127-1

**Published:** 2022-02-16

**Authors:** E. Erickson, H. M. Patch, C. M. Grozinger

**Affiliations:** Department of Entomology, Center for Pollinator Research, Huck Institutes of the Life Sciences, Pennsylvania State University, University Park, USA

Correction to: *Scientific Reports* 10.1038/s41598-021-95892-w, published online 30 August 2021

The original version of this Article contained an error in the Data Availability section, where the Dryad database URL was incorrect.

“All data and R scripts relating to this research has been made publicly available via Dryad https://doi.ord/10.5061/dray.s4mw6m96s.”

now reads:

“All data and R scripts relating to this research have been made publicly available via Dryad 10.5061/dryad.s4mw6m96s.”

The original Article has been corrected.

