# Supplementary material for: Herbaceous perennial ornamental plants can support complex pollinator communities
Source: Sci Rep. 2021 Aug 30;11:17352. doi: 10.1038/s41598-021-95892-w (PMC8405689; doi:10.1038/s41598-021-95892-w)
Supplement: Supplementary file 1 — Supplementary Information. [file 41598_2021_95892_MOESM1_ESM.pdf]

Supplementary Materials

Herbaceous perennial ornamental plants can support complex pollinator communities

Erickson, E.[1]*, Patch, H.M[1], Grozinger C.M.[1]

[1]Department of Entomology, Center for Pollinator Research, Huck Institutes of the Life Sciences, Pennsylvania State University

Table S1. Bee species collected in traps and on plants

Bee Species	*# on plants*	*# in traps*	*Diet*	*Parasitism*	*Reference*
Agapostemon sericeus (Forster, 1771)	2	1	P	N	Bartomeus et al., 2013
Agapostemon virescens (Fabricius, 1775)	4	2	P	N	Bartomeus et al., 2013
Andrena nuda (Robertson, 1891)	0	1	P	N	Bartomeus et al., 2013
Andrena chromotricha (Cockerell, 1899)	1	0	P	N	Discover Life
Andrena hippotes (Robertson, 1895)	0	1	P	N	Discover Life
Andrena imitatrix/morrisonella	1	0	P	N	Bartomeus et al., 2013
Andrena mandibularis (Robertson, 1892)	0	1	P	N	Bartomeus et al., 2013
Andrena nasonii (Robertson, 1895)	0	4	P	N	Bartomeus et al., 2013
Andrena perplexa (Smith, 1853)	0	3	P	N	Bartomeus et al., 2013
Andrena wilkella (Kirby, 1802)	4	2	P	N	Savoy-Burke, 2017
Anthidium manicatum (Linnaeus, 1758)	4	2	P	N	Payne et al., 2011
Anthophora bomboides (Kirby, 1838)	9	20	P	N	Rykken, 2017
Anthophora terminalis (Cresson, 1869)	13	3	P	N	Bartomeus et al., 2013
Anthophora abrupta (Say, 1838)	1	0	P	N	Norden, 1984
Apis mellifera (Linnaeus, 1758)	142	12	P	N	Discover Life
Augochlora pura (Say, 1837)	735	19	P	N	Bartomeus et al., 2013
Augochlorella aurata (Smith, 1853)	65	0	P	N	Bartomeus et al., 2013
Augochloropsis m. fulgida (Smith, 1853)	2	0	P	N	Bartomeus et al., 2013
Bombus bimaculatus (Cresson, 1863)	213	21	P	N	Bartomeus et al., 2013
Bombus citrinus (Smith, 1854)	1	0	NA	Y	Discover Life
Bombus fernaldae (Franklin, 1911)	2	0	NA	Y	Discover Life
Bombus fervidus (Fabricius, 1798)	0	2	P	N	Bartomeus et al., 2013
Bombus fervidus/pensylvanicus	0	2	P	N	Bartomeus et al., 2013
Bombus griseocollis (De Greer, 1773)	60	3	P	N	Bartomeus et al., 2013
Bombus impatiens (Cresson, 1863)	809	41	P	N	Bartomeus et al., 2013
Bombus perplexus (Cresson, 1863)	29	0	P	N	Bartomeus et al., 2013
Bombus sandersoni (Franklin, 1913)	0	3	P	N	Bartomeus et al., 2013
Bombus ternarius (Say, 1837)	0	1	P	N	Bartomeus et al., 2013
Bombus terricola (Kirby, 1837)	0	2	P	N	Bartomeus et al., 2013
Bombus vagans (Smith, 1854)	117	17	P	N	Bartomeus et al., 2013
Bombus spp.	6	0	NA	NA	
Ceratina calcerata (Roberston, 1900)	472	7	P	N	Bartomeus et al., 2013
Ceratina dupla (Say, 1837)	34	2	P	N	Bartomeus et al., 2013
Ceratina mikmaqi (Rehan & Sheffield, 2011)	62	3	P	N	Bartomeus et al., 2013
Ceratina strenua (Smith, 1879)	10	1	P	N	Bartomeus et al., 2013
Ceratina spp.	18	0	NA	NA	Discover Life
Coelioxys alternatus (Say, 1837)	1	0	NA	Y	Discover Life
Coelioxys sayi (Robertson, 1897)	1	0	NA	Y	Discover Life
Eucera atriventris (Smith, 1854)	1	0	P	N	Savoy-Burke, 2017
Halictus confusus (Smith, 1853)	15	3	P	N	Bartomeus et al., 2013
Halictus ligatus (Say, 1837)	119	6	P	N	Bartomeus et al., 2013
Halictus rubicundus (Christ, 1791)	26	2	P	N	Bartomeus et al., 2013
Heriades carinatus (Cresson, 1864)	4	0	P	N	Bartomeus et al., 2013
Heriades leaviti/variolosus	1	0	P	N	Discover Life
Hoplitis pilosifrons (Cresson, 1864)	1	0	P	N	Bartomeus et al., 2013
Hoplitis producta (Cresson, 1864)	0	1	P	N	Bartomeus et al., 2013
Hylaeus affinis/modestus	62	3	P	N	Bartomeus et al., 2013
Hylaeus annulatus (Linnaeus, 1758)	1	0	P	N	Discover Life
Hylaeus modestus (Say, 1837)	2	0	P	N	Bartomeus et al., 2013
Lasioglossum abanci (Crawford, 1932)	1	0	P	N	Discover Life
Lasioglossum admirandum (Sandhouse, 1924)	2	0	P	N	Discover Life
Lasioglossum athabascence (Sandhouse, 1933)	1	0	P	N	Discover Life
Lasioglossum birkmanni (Crawford, 1906)	0	1	P	N	Normandin et al., 2017
Lasioglossum bruneri (Crawford, 1902)	1	3	P	N	Bartomeus et al., 2013
Lasioglossum catellae (Ellis, 1913)	1	0	P	N	Discover Life
Lasioglossum cinctipes (Provancher, 1888)	1	0	P	N	Bartomeus et al., 2013
Lasioglossum coriaceum (Smith, 1853)	10	7	P	N	Bartomeus et al., 2013

Lasioglossum cressonii (Robertson, 1890)	3	3	P	N	Bartomeus et al., 2013
Lasioglossum dreisbachi (Mitchell, 1960)	1	0	P	N	Nardone, 2013
Lasioglossum foxii (Robertson, 1895)	2	0	P	N	Bartomeus et al., 2013
Lasioglossum fuscipenne (Smith, 1853)	0	1	P	N	Discover Life
Lasioglossum gotham (Gibbs, 2011)	1	0	P	N	Discover Life
Lasioglossum heterognathum (Mitchell, 1960)	1	0	P	N	Bartomeus et al., 2013
Lasioglossum hitchensi (Gibbs, 2012)	14	8	P	N	Rykken, 2017
Lasioglossum leucozonium (Schrank, 1781)	1	2	P	N	Bartomeus et al., 2013
Lasioglossum lineatulum (Crawford, 1906)	0	1	P	N	Bartomeus et al., 2013
Lasioglossum laevissimum (Smith, 1853)	1	0	P	N	Discover Life
Lasioglossum obscurum (Robertson, 1892)	2	0	P	N	Bartomeus et al., 2013
Lasioglossum oceanicum (Cockerell, 1916)	0	5	P	N	Lerman & Milam, 2016
Lasioglossum paradmirandum (Knerer & Atwood, 1966)	1	0	P	N	Rykken, 2017
Lasioglossum pectorale (Smith, 1853)	13	6	P	N	Bartomeus et al., 2013
Lasioglossum pilosum (Smith, 1853)	0	1	P	N	Bartomeus et al., 2013
Lasioglossum quebecense (Crawford, 1907)	2	1	P	N	Bartomeus et al., 2013
Lasioglossum trigeminum (Gibbs, 2011)	4	3	P	N	Seitz et al., 2020
Lasioglossum truncatum (Robertson, 1901)	5	57	P	N	Bartomeus et al., 2013
Lasioglossum versans (Lovell, 1905)	3	0	P	N	Bartomeus et al., 2013
Lasioglossum versatum (Robertson, 1902)	8	19	P	N	Bartomeus et al., 2013
Lasioglossum viridatum (Lovell, 1905)	1	0	P	N	Normandin et al., 2017
Lasioglossum viridatum grp.	0	1			
Lasioglossum weemsi (Mitchell, 1960)	1	2	P	N	Bartomeus et al., 2013
Lasioglossum zephyrum (Smith, 1853)	0	3	P	N	Bartomeus et al., 2013
Lasioglossum spp.	7	7	NA	NA	
Megachile campanulae (Robertson, 1903)	11	0	P	N	Bartomeus et al., 2013
Megachile concinna (Smith, 1879)	1	0	P	N	Discover Life
Megachile frugalis (Cresson, 1872)	1	0	P	N	Discover Life
Megachile gemula (Cresson, 1878)	2	0	P	N	Bartomeus et al., 2013
Megachile mendica (Cresson, 1878)	8	1	P	N	Bartomeus et al., 2013
Megachile montivaga (Cresson, 1878)	1	0	P	N	Discover Life
Megachile pugnata (Smith, 1837)	8	0	O	N	Bartomeus et al., 2013
Megachile relativa (Cresson, 1878)	2	0	P	N	Bartomeus et al., 2013
Megachile rotundata (Fabricius, 1787)	0	1	P	N	Discover Life
Mecgachile sculpturalis (Smith, 1853)	3	0	P	N	Le Féon et al., 2018
Melissodes bimaculatus (Lepeletier, 1825)	3	20	P	N	Bartomeus et al., 2013
Melissodes denticulata (Smith, 1854)	5	0	O	N	Fowler, 2016
Melissodes desponsa (Smith, 1854)	6	16	O	N	Fowler, 2016
Melissodes druriellus (Kirby, 1802)	1	0	O	N	Fowler, 2016
Melissodes subillatus (LaBerge, 1961)	1	0	O	N	Fowler, 2016
Melissodes trinodis (Robertson, 1901)	7	8	O	N	Fowler, 2016
Melissodes spp.	0	0	NA	NA	
Nomada illinoensis (Robertson, 1900)	1	0	NA	Y	Lerman & Milam, 2016
Nomada vincta (Say, 1837)	0	1	NA	Y	Alexander, 1991
Osmia atriventris (Cresson, 1864)	1	0	P	N	Bartomeus et al., 2013
Osmia bucephala (Cresson, 1864)	9	1	P	N	Bartomeus et al., 2013
Osmia felti (Cockerell, 1911)	1	0	P	N	Bartomeus et al., 2013
Osmia pumila (Cresson, 1864)	3	1	P	N	Bartomeus et al., 2013
Peponapis pruinosa (Say, 1837)	1	93	O	N	Bartomeus et al., 2013
Triepeolus donatus (Smith, 1854)	1	1	NA	Y	Discover Life
Triepeolus lunatus (Say, 1824)	1	0	NA	Y	Discover Life
Triepeolus remigatus (Fabricius, 1804)	1	0	NA	Y	Discover Life
Xylocopa virginica (Linnaeus, 1771)	85	2	P	N	Bartomeus et al., 2013

Table S2. Background plant species identifications (2019)

Plant Species	***# individual flowers at Site 1***	***# individual flowers at Site 2***
Achillea millefolium (L.)	0	19
Arctium spp.	0	3
Bidens cernua (L.)	5	0
Cirsium spp.	172	7
Conoclinium coelestinum (L.) DC.	0	20
Daucus carota (L.)	486	591
Epilobium coloratum (Biehler)	2	0
Erigeron annus (L.) Pers.	1629	859
Eupatorium serotinium (Michx.)	15	0
Euthamia gramifolia (L.) Nutt	3	0
Galium spp.	1293	0
Geum spp.	55	0
Glechoma hederacea (L.)	0	9
Hypericum punctatum (Lam. 1796)	6	0
Leucanthemum spp.	0	15
Lingustrum spp.	200	0
Linaria spp.	0	0
Lobelia spicata (Lamarck)	11	1
Medicago lupulina (L.)	0	4
Monarda spp.	0	3
Oxalis spp.	0	46
Penstemon digitalis (Nutt. ex. Sims)	15	0
Plantago lanceolata (L.)	0	917
Polygonum sagittatum (L.) H. Gross 1919	160	914
Polygonum spp.	6	0
Potentilla spp.	0	24
Securigera varia (L.) Lassen	0	1300
Senna hebecarpa (Fernald) H.S. Irwin & Barneby	0	13
Sisyrinchium spp.	0	1
Solidago spp.	1368	0
Stellaria graminea (L.)	809	110
Symphyotrichum lateriflorum (L.) Á.Löve & D.Löve	190	0
Symphyotrichum novae-angliae (L.) G.L. Nesom	0	25
Symphyotrichum pilosum (Willd) G.L. Nesom	855	2895
Taraxacum spp.	1	1
Trifolium pratense (L.)	0	390
Trifolium repens (L.)	256	505
Triglochin spp.	0	1

Table S3. Morphotaxa groupings for field observations and corresponding classification used for analyses

Apis mellifera	Anthophila	Small syrphid	Diptera
Bombus spp.	Anthophila	Bombyliidae spp.	Diptera
Hylaeus spp.	Anthophila	*Epargyreus clarus*	Lepidoptera
Small dark sweat bee	Anthophila	Hawkmoth	Lepidoptera
Ceratina spp.	Anthophila	*Danaus plexxipus*	Lepidoptera
Andrenidae spp.	Anthophila	Swallowtail	Lepidoptera
Megachilidae spp.	Anthophila	Unk. lepidoptera	Lepidoptera
Cuckoo Bee	Anthophila	Muscidae spp.	Diptera
Green sweat bee	Anthophila	Calliphoridae spp.	Diptera
Melissodes spp.	Anthophila	Unk. diptera	Diptera
Anthophora spp.	Anthophila	Soldier Beetle	Coleoptera
Xylocopa virginica	Anthophila	Flower Beetle	Coleoptera
Pieridae spp.	Lepidoptera	Unk. Coleoptera	Coleoptera
Large syrphid	Diptera	Unk. Wasp	Non-anth. Hym.

Observed visitor abundance model

glmer(value ~ offset(log(sampTime)) + Cultivar + Site + Cultivar:Site + Year + scaled(floral area) + Cultivar:Year + Year:Site + (1|plant replicate) + (1|Observer), control=glmerControl(optimizer="bobyqa", optCtrl = list(maxfun = 400000)), family = poisson(link = log), data = Field_obs_abundance_analysis4)

GVIF^(1/(2*Df)) (calculated in the 'car' Package [14])
Cultivar: 2.275187
Site: 4.109522
Year: 4.152318
scaled(floral area): 1.269113
Cultivar:Site: 1.791212
Cultivar:Year: 1.711395
Site:Year: 2.853588

AIC: 6924.7
Scaled residuals:
Min: -5.7167 1Q: -1.0174 Median: -0.5075 3Q: 0.7670 Max: 12.2911

Fertilization rates for plants in the field and the greenhouse
Osmocote 15-8-11 tablets
Echinacea and Rudbeckia: 2 tablets
Salvia, Nepeta, Agastache: 3 tablets

Figure S1: Field plot design

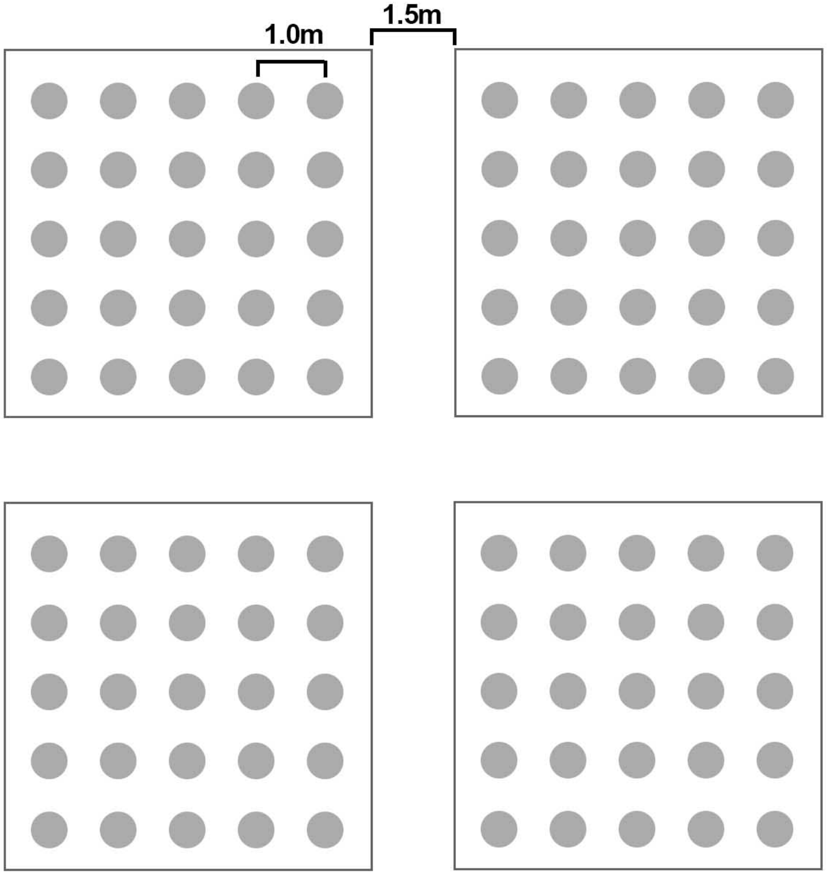

Figure S1: Plots were arranged in a Randomized Complete Block Design with four blocks per plot and one replicate of each of the 25 cultivars per block, for a total of eight replicates per cultivar across two sites.

References

1. Bartomeus, I. *et al.* Historical changes in northeastern US bee pollinators related to shared ecological traits. *Proc. Natl. Acad. Sci. U. S. A.* **110**, 4656–60 (2013).
2. Discover Life.
3. Savoy-Burke, G. Woodland bee diversity in the Mid-Atlantic. (University of Delaware, 2017).
4. Payne, A., Schildroth, D. A. & Starks, P. T. Nest site selection in the European wool-carder bee, *Anthidium manicatum*, with methods for an emerging model species. *Apidologie* **42**, 181–191 (2011).
5. Jessica J. Rykken. *Catch the buzz-pollinator diversity, distribution, and phenology in Shenandoah National Park*. (2017).
6. Norden, B. Nesting biology of *Anthophora abrupta* (Hymenoptera: Anthophoridae). *J. Kansas Entomol. Soc.* **57**, 243–262 (1984).
7. Normandin, É., Vereecken, N. J., Buddle, C. M. & Fournier, V. Taxonomic and functional trait diversity of wild bees in different urban settings. *PeerJ* **5**, e3051 (2017).
8. Nardone, E. The bees of Algonquin Park: a study of their distribution, their community guild

structure, and the use of various sampling techniques in kogged and unlogged hardwood stands. (University of Guelph, 2013).

9. Lerman, S. B. & Milam, J. Bee Fauna and floral abundance within lawn-dominated suburban yards in Springfield, MA. *Ann. Entomol. Soc. Am.* **109**, 713–723 (2016).
10. Seitz, N., VanEngelsdorp, D. & Leonhardt, S. D. Are native and non-native pollinator friendly plants equally valuable for native wild bee communities? *Ecol. Evol.* **10**, 12838–12850 (2020).
11. Le Féon, V. *et al.* Range expansion of the Asian native giant resin bee *Megachile sculpturalis* (Hymenoptera, Apoidea, Megachilidae) in France. *Ecol. Evol.* **8**, 1534–1542 (2018).
12. Fowler, J. Specialist bees of the Northeast: host plants and habitat conservation. *Northeast. Nat.* **23**, 305–320 (2016).
13. Alexander, B. *Nomada* phylogeny reconsidered (Hymenoptera: Anthophoridae). *J. Nat. Hist.* **25**, 315–330 (1991).
14. Fox, J. & Weisberg, S. *An {R} Companion to Applied Regression*. (Sage, 2019).
15. R Core Team. R: A language and environment for statistical computing. https://www.R-project.org/. (R Foundation for Statistical Computing, 2020)
